# Pharmacological Modulation of Vagal Nerve Activity in Cardiovascular Diseases

**DOI:** 10.1007/s12264-018-0286-7

**Published:** 2018-09-14

**Authors:** Longzhu Liu, Ming Zhao, Xiaojiang Yu, Weijin Zang

**Affiliations:** Department of Pharmacology, School of Basic Medical Sciences, Xi’an Jiaotong University Health Science Center, Xi’an, 710061 China

**Keywords:** Cardiovascular disease, Cholinergic drugs, Muscarinic receptor, α7-nACh receptor, Vagus nerve, Vagomimetic drugs

## Abstract

Cardiovascular diseases are life-threatening illnesses with high morbidity and mortality. Suppressed vagal (parasympathetic) activity and increased sympathetic activity are involved in these diseases. Currently, pharmacological interventions primarily aim to inhibit over-excitation of sympathetic nerves, while vagal modulation has been largely neglected. Many studies have demonstrated that increased vagal activity reduces cardiovascular risk factors in both animal models and human patients. Therefore, the improvement of vagal activity may be an alternate approach for the treatment of cardiovascular diseases. However, drugs used for vagus nerve activation in cardiovascular diseases are limited in the clinic. In this review, we provide an overview of the potential drug targets for modulating vagal nerve activation, including muscarinic, and β-adrenergic receptors. In addition, vagomimetic drugs (such as choline, acetylcholine, and pyridostigmine) and the mechanism underlying their cardiovascular protective effects are also discussed.

## Introduction

The vagus nerve, which is the longest cranial nerve in the human body, innervates the thoracic and abdominal organs, including the autonomic, cardiovascular, respiratory, gastrointestinal, immune, and endocrine systems. The vagus nerve is a key component of the autonomic nervous system (ANS) containing 80% afferent and 20% efferent nerve fibers [1]. The vagal afferent fibers sense a range of stimuli, including pressure, pain, stretch, temperature, chemicals, osmotic pressure, and inflammation. These sensory signals are gathered at the vagal nuclei and are transmitted to multiple brain regions. After being processed in the brain, regulatory signals are transmitted by the vagal efferent fibers. Vagal efferent fibers, which arise from the nucleus ambiguus and the dorsal motor nucleus, are primarily cholinergic, using acetylcholine (ACh) as their major neurotransmitter. Imbalances in the ANS, which are characterized by a reduction in vagal tone accompanied by increased sympathetic activity, are associated with disease progression and negative clinical outcomes, triggering many cardiovascular diseases, such as heart failure [2], arrhythmia [3] and hypertension [4]. Many studies have demonstrated that increased vagal activity reduces cardiovascular risk factors in both animal models and human patients [5–8]. However, the roles of drugs that activate vagal nerves and vagomimetic drugs in the treatment of cardiovascular disease are still not fully understood.

In the present review, we summarize the recent studies detailing the drugs used in vagal nerve activation for the treatment of cardiovascular disease, as well as putative mechanisms underlying the cardiovascular protective effects of these compounds.

## Alteration of Vagal Tone in Cardiovascular Disease

The heart is dominated by both sympathetic and parasympathetic (vagal) nerves. The innervation by sympathetic and vagal nerves is asymmetrical. The right sympathetic and vagal nerves innervating the sinus node and atrium, primarily control the heart rate; the left sympathetic and vagal nerves innervating the atrioventricular junction and the left ventricle, primarily control myocardial contractility and cardiac output [9]. The vagus nerve plays a vital role in maintaining normal cardiovascular function. Under physiological conditions, the sympathetic and parasympathetic (vagal) activities modulating cardiac function undergo a reciprocal regulation, leading to sympathovagal balance [10]. The sympathetic neurotransmitter catecholamine is released, which increases the heart rate and myocardial contractility [11]. Simultaneously, the vagal nerve releases ACh to reduce the conduction of pacemaker cells in the sinus node, thereby lowering the heart rate and the myocardial contractility [12]. Under pathological conditions, autonomic imbalance and increased cardiac sympathetic nerve activity lead to an increased heart rate, increased myocardial contractility, and increased myocardial oxygen consumption. Meanwhile, decreased vagal nerve discharge and a decrease in the amount of ACh released into the synaptic cleft, lead to the loss of the ability of the vagal nerve to reduce the heart rate and decrease myocardial contractility. Ultimately, this loss results in cardiac overload and enhanced cardiovascular damage [4]. Thus, the restoration or enhancement of vagal nerve activity may be a promising therapy for cardiovascular disease.

The activation of vagal nerves occurs at multiple levels, including the afferent, central, and efferent components and all related effectors. Our primary concern is the relationship between activation of the vagal efferent component and cardiovascular disease. Vagal efferents can be activated during the following processes: (1) an increase in neuronal discharge and ACh release by activation of the central axis and direct vagal nerve stimulation; (2) the administration of cholinergic drugs or the indirect increase in ACh levels by administration of cholinesterase inhibitors; and (3) an increase in ACh bioavailability by activation of the ACh-associated receptors (M receptors and the N receptor) and downstream pathways.

**Fig. 1:**
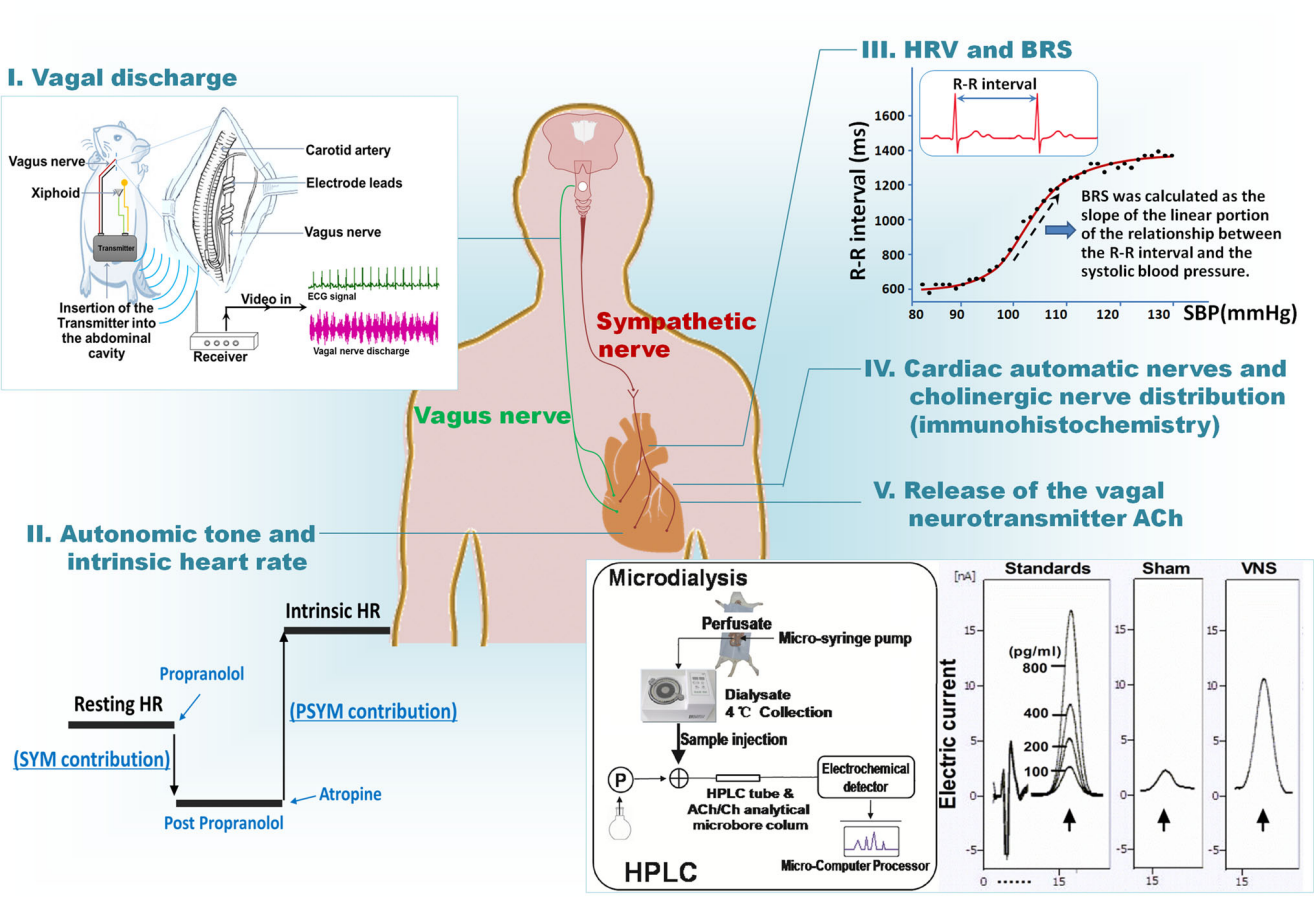
Methods and indices used to evaluate vagal activity in cardiovascular disease. Vagus activity can be evaluated by (I) measurement of the vagal discharge (image adapted from Lu Y *et al.* [27]); (II) autonomic tone and intrinsic heart rate; (III) HRV and BRS; (IV) cardiac autonomic nerves and cholinergic nerve distribution; and (V) detection of the release of ACh into the circulation (image adapted from Zhao M *et al.* [6]). ACh, acetylcholine; BRS, baroreflex sensitivity; HPLC, high performance liquid chromatography; HRV, heart rate variability; PSYM, parasympathetic; SYM, sympathetic.

Although many methods can be used to study vagal activity in experiments, only heart rate variability (HRV) and baroreflex sensitivity (BRS) are commonly used in the clinic [13]. HRV and BRS are important markers that are typically used to indirectly assess vagal activity [14–21]. BRS is primarily regarded as a measure of vagal reflex activity and is expressed in milliseconds of increase in the RR interval consequent to an increase of 1 mmHg in blood pressure [22]. HRV is the variation over time of the period between consecutive heartbeats [23]. Other methods, such as the ACh level in the blood measured by microdialysis [6], cardiac automatic nerves and cholinergic nerve distribution by immunohistochemistry [24], autonomic tone and intrinsic heart rate by administration of atropine and propranolol [25, 26] or neuronal/vagal discharge measured by a biological signal analytical system [27], can also be used to evaluate vagal activity in experiments (Fig. 1).

## Decreased Vagal Tone is Associated with Increased Mortality in Heart Failure

Heart failure (HF) is a leading cause of morbidity and mortality. The ANS plays an important role in regulating pathological cardiac remodeling in HF. An imbalance in the ANS is a predictor for a negative prognosis in patients with HF. It has been demonstrated that decreased vagal tone is associated with an increase in mortality for HF [28]. A recent report indicates that the neurotransmitter response of the cardiac vagal neurons in the brain stem is altered [29]. The sympathovagal imbalance often results in left ventricular dysfunction during the early stages of HF [30]. Decreased ventricular epicardial vagal nerve density has also been reported to potentially contribute to impaired cardiac vagal control in rats with HF [31]. For the treatment of HF, vagus nerve stimulation (VNS) has been shown to be beneficial for improving the autonomic balance in HF by enhancing vagal tone [32]. A recent report has also shown that short-term intravenous VNS markedly reduces infarct size and prevents subsequent HF. The bradycardic effect may play an important role in the beneficial effect of VNS [33].

### Arrhythmia with Reduced Vagal Tone after Myocardial Infarction

Many studies have indicated that the ANS plays a pivotal role in the development of arrhythmias [34–36]. Electrical wave-break-induced re-entry can cause the onset of arrhythmia. Wave-break is primarily affected by two factors. Anatomical factors, such as scars and fibrosis, affect wave-break and lead to tissue heterogeneity and electrical remodeling. Changes in membrane voltage and intracellular Ca^2+^ levels also affect wave-break. Arrhythmia with reduced vagal tone after myocardial infarction is a life-threatening risk factor [13]. We have reported that cholinergic nerves and M2 receptors are located in both rat atria and ventricles [37]. Inagaki *et al.* have found that VNS reduces the rate of ventricular fibrillation from 62% to 7% in cats [38]. Over the years, VNS has been shown not only to slow down the sinus rate but also to display many beneficial effects that protect the heart against ventricular arrhythmias, including decreased heart rate, reduced atrioventricular conduction, and reduced atrial contraction [39]. Vagal modulation plays a significant role in the initiation and development of arrhythmias, and novel vagomimetic drug discovery may hold the key to treatment.

### Reduced Vagal Activity Contributes to Hypertensive Organ Damage

The ANS plays a critical role in maintaining cardiovascular homeostasis during hypertension. Vagal nerve fibers can modulate local and systemic inflammatory responses, known as the ‘cholinergic anti-inflammatory pathway’ [40]. Both a decrease in ACh release and dysfunction of the α7 nicotinic ACh receptor contribute to hypertensive organ damage. Recently, chronic VNS has been reported to prevent hypertension-induced endothelial dysfunction and aortic stiffening in spontaneously hypertensive rats (SHRs) [41]. Our group also demonstrated that an increase in the expression of TLR4 and pro-inflammatory cytokines may be correlated with suppressed vagal activity in SHRs [16]. Therefore, increasing vagal activity may be an interesting alternative approach for antihypertensive therapy.

Abnormal ANS activity is correlated with many other cardiovascular diseases, such as myocardial infarction [42], ischemia/reperfusion injury [43], and atherosclerosis [44]. In summary, the vagal nerve plays an important role in many cardiovascular diseases, and further investigation is required to find therapies for restoring impaired vagal nerve function to improve clinical outcomes and prognoses.

## Potential Targets for Vagal Nerve Activation in Cardiovascular Disease

### Muscarinic Receptors and Cardiovascular Diseases

Cholinergic receptors are conventionally divided into two types of ACh receptors (AChRs), namely, the muscarinic and nicotinic receptors. The muscarinic ACh receptor (mAChR) is a member of a sub-family of G protein-coupled receptors that includes the M1–M5 subtypes [45]. These mAChRs exist in many tissues and cells, acting under the control of parasympathetic innervation. Among these mAChRs, the M2 and M3 receptor subtypes are important in the cardiovascular system. Previous studies have shown that the M2 receptor is primarily distributed in myocardial cells [46]. Our studies have also shown that ACh inhibits tumor necrosis factor alpha production by downregulating p38 mitogen-activated protein kinase (MAPK) and JNK phosphorylation; this is mediated by the M2 receptor, and M2 antagonists or the knockdown of M2 receptor expression by siRNA abolishes the effects of ACh-induced protection in cardiomyocytes [47, 48]. Recent studies have found that the M3 receptor subtype is also distributed in myocardial cells [49], and plays a protective role in cardiovascular disease [50]. Some studies have reported that M3 receptor activation reduces angiotensin II-induced cardiac hypertrophy, corrects cardiac hemodynamic dysfunction, inhibits myocardial cell apoptosis, and reduces myocardial injury [51]. A recent study indicated that activation of the M3 receptor by VNS improves mitochondrial dynamics and mitochondrial function in isoproterenol-induced myocardial ischemia [52]. The pharmacological activation of M3 receptors also has a cardioprotective effect in ischemia-induced ischemia/reperfusion (I/R) injury [7]. Furthermore, the overexpression of M3 receptors decreases the incidence of arrhythmias in a mouse model of myocardial I/R injury [53].

### Nicotinic Acetylcholine Receptors and Cardiovascular Diseases

Nicotinic ACh receptors (nAchRs) are ion channels formed by five subunits delimiting a central aqueous pore. nAchRs have been identified in many cell types, including vascular smooth muscle cells [54] and endothelial cells [55]. nAchRs have been suggested to be involved in the cardioprotection conferred by vagal nerve stimulation [14]. In particular, the primary function of the α7nAchR subtype in mediating this inflammatory response is now well-accepted [56]. ACh binds to the α7nAchR, which inhibits the transcriptional activity of p38 MAPK and nuclear factor-kappa B [57]. Furthermore, α7nAchR activation can also recruit Janus kinase 2 (Jak2) to form a heterodimeric complex, initiating an intracellular transduction response mediated by signal transducer and activator of transcription 3 (STAT3) [58]. Vagal stimulation protects against myocardial I/R-induced remote vascular dysfunction through the cholinergic anti-inflammatory pathway, which is dependent upon α7nACh receptors [6]. Previous studies have also shown that α7nACh receptors block inflammasome complex activation by suppressing mitochondrial DNA release [59]. Therefore, the pharmacological activation of α7nAchR, by improving vagal activity, characterizes an alternative approach for treating cardiovascular disease.

### β2 Adrenergic Receptors and Cardiovascular Disease

Adrenergic receptors (ARs) are a large family of seven-transmembrane domain receptors responsive to catecholamines. ACh released by the vagus nerve in the celiac mesenteric ganglia activates the splenic nerve through postsynaptic α7nAchRs [60]. Then, noradrenaline released from splenic nerve terminals promotes β2AR activation in T cells in the spleen [61]. The β2AR-activated T cells then produce ACh, which activates α7nAchRs on cytokine-producing macrophages, thus inhibiting the release of cytokines from the spleen [62]. These studies have indicated that β2AR-activated T cells in the spleen are critical for the anti-inflammatory action of the vagus nerve [63], and β2ARs represent a potential pharmacological target for vagal activation in cardiovascular disease.

## Drugs for Cardioprotection by Vagal Nerve Activation

Modulation of the vagal nerve activity is theoretically a promising therapy for cardiovascular disease. Current, major, non-drug-activated vagal nerve activation approaches include direct vagal stimulation through surgically implanted stimulators [64], and indirect renal sympathectomy [65], aerobic exercise, and yoga [66]. At present, VNS has been clinically applied in patients with drug-resistant epilepsy, depression, and heart failure. VNS has also been found to have a protective effect against myocardial infarction-induced arrhythmias, inhibit the abnormal ventricular electrophysiological changes that can be induced by sympathetic over-activation, and reduce the incidence of heart fibrillation [64].

**Fig. 2:**
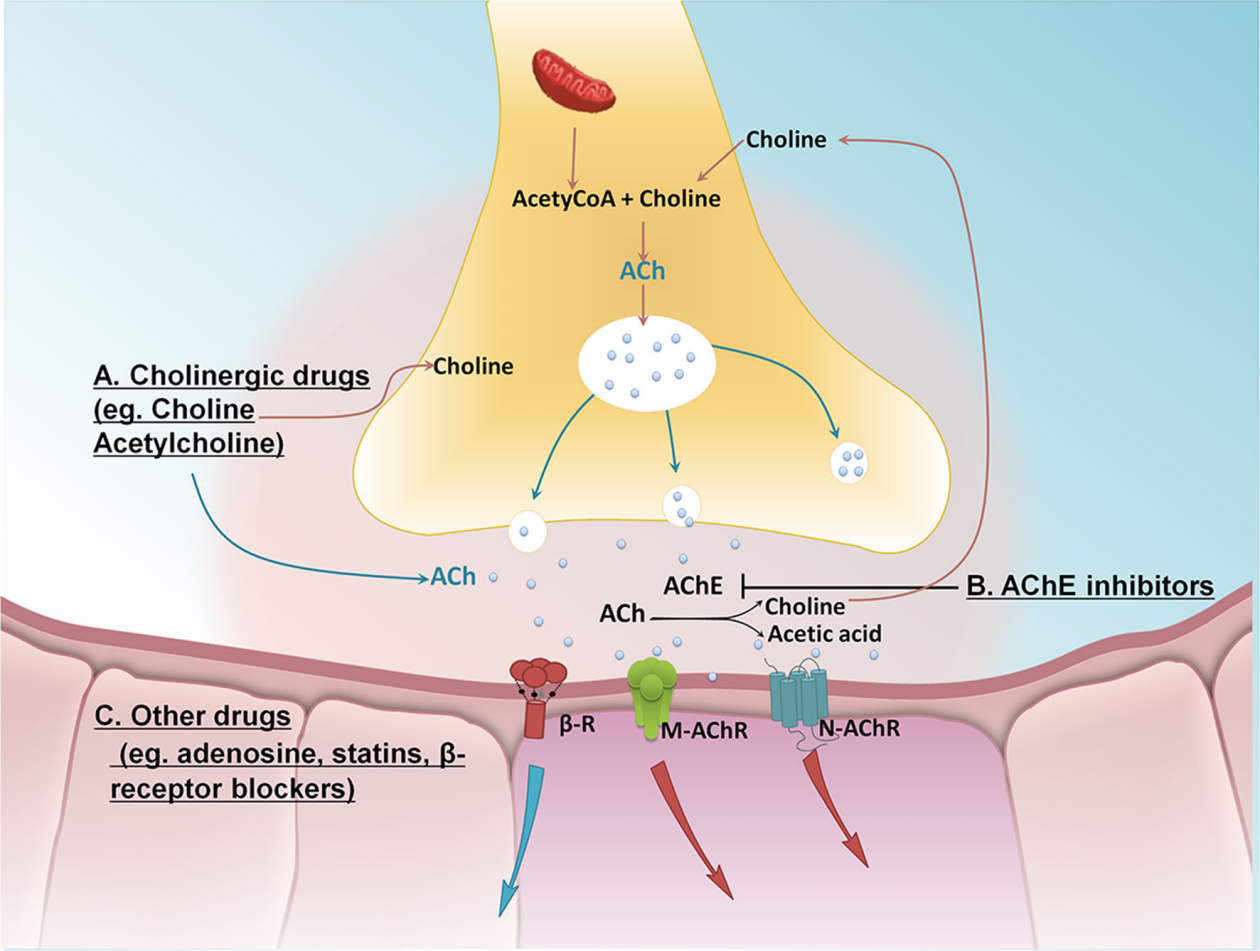
Schematic of the drug targets for vagal nerve activation. **A** The vagal transmitter ACh is synthesized *in vivo* by the catalysis of choline and acetyl-CoA by choline acetyltransferase, thus directly increasing the levels of synaptic ACh [67]. **B** Cholinesterase inhibitors increase the levels of synaptic ACh by decreasing its degradation, and thus directly increasing vagal tone [68]. **C** Adenosine [69], statins [70], beta-receptor blockers [71], and ACEIs [72] indirectly activate vagal tone by acting on the related receptors. Acetyl-CoA, Acetyl-coenzyme A; AChE, acetylcholine esterase; ACEIs, angiotensin-converting-enzyme inhibitors; ACh, acetylcholine.

Although there is already much evidence that direct VNS can effectively improve the occurrence and development of cardiovascular disease [52, 64], due to surgical trauma, poor patient compliance, and an inadequate response in patients with cardiac insufficiency, its widespread application in the clinic has been limited. Therefore, activation of the vagus nerve by pharmacological tools may be another option for patients with cardiovascular disease as an alternative to direct VNS. The primary drug targets used for vagal nerve activation in cardiovascular diseases have included: (1) vagomimetic agents that directly increase the levels of synaptic ACh to activate muscarinic and nicotinic receptors; (2) cholinesterase inhibitors that indirectly decrease the degradation of ACh; and (3) adenosine, statins, beta-receptor blockers and angiotensin-converting-enzyme inhibitors (ACEIs), which also indirectly activate vagal tone; however, the underlying mechanisms through which these drugs stimulate vagal activation still require further investigation (Fig. 2).

## Cholinergic Drugs Used in the Treatment of Cardiovascular Diseases

### Choline

The essential nutrient choline participates in various biological processes. It is a component of two biologically important molecules, phosphatidylcholine and ACh. Choline has been used in the clinical treatment of steatohepatitis [73]. Accumulating evidence has demonstrated that choline also has multiple protective effects against various cardiovascular diseases, including myocardial infarction [42], arrhythmias [74], cardiac hypertrophy [75, 76], and I/R injury [77]. Choline has also been demonstrated to have a protective effect against vascular damage in rats after I/R through the inhibition of the reactive oxygen species (ROS)-mediated Ca^2+^/calmodulin-dependent protein kinase II (CaMKII) pathway and the regulation of Ca^2+^cycling proteins. These protective effects may be attributed to activation of the M3 receptor [7]. Recently, our group demonstrated that choline slows the progress of hypertension and ameliorates cardiac, renal, and vascular damage in SHRs. These protective effects may be related to the improvement in vagal activity and the reduction in inflammatory cytokines found in SHRs [16]. These studies indicate that choline may be a promising therapeutic agent for the alleviation of cardiovascular damage in cardiovascular disease, although the mechanisms underlying its cardiovascular protective effects require further investigation.

### Acetylcholine

ACh is the endogenous agonist for the two primary types of cholinoceptor, the muscarinic and nicotinic receptors and they will likely become novel targets for drug development [78]. *In vivo* microdialysis studies have demonstrated that vagal stimulation increases the ACh concentration in the mesenteric circulation, suggesting that the ACh released from the vagus nerve is transported into the mesenteric circulation and has effects on endothelial cells and vascular smooth muscle cells [6]. Studies in our laboratory have demonstrated that ACh prevents angiotensin II-induced cardiomyocyte injury through down-regulation of the angiotensin II type 1 receptor and inhibition of ROS-mediated p38 MAPK activation, as well as regulation of Bcl-2, Bax, and caspase-3 expression [48, 79–81]. Recently, we demonstrated that ACh markedly suppresses the mitochondrial unfolded protein response and endoplasmic reticulum stress, which alleviate I/R injury [82, 83]. ACh may also modulate the abnormal inter-organelle crosstalk in cardiovascular diseases. Our studies indicated that ACh attenuates intracellular Ca^2+^ overload by inhibiting the formation of the VDAC1/Grp75/IP3R1 complex and the NCX1-TRPC3-IP3R1 complex in mitochondria-endoplasmic reticulum and endoplasmic reticulum-plasma membrane connection sites in human umbilical vein endothelial cells, indicating that the inhibition of inter-organelle crosstalk may be a mechanism for vagal protective effects in cardiovascular disease [84, 85]. Currently, ACh is primarily used as a pharmaceutical tool due to the multiple effects that it has throughout the body.

## Acetylcholinesterase Inhibitors for the Treatment of Cardiovascular Disease

### Pyridostigmine

There are two types of cholinesterase inhibitors: reversible anticholinesterase and irreversible cholinesterase inhibitors. Irreversible cholinesterase inhibitors are used as organic pesticides and are highly toxic, while reversible anticholinesterases have relatively lower toxicity. Peripheral reversible acetylcholinesterase inhibitors such as pyridostigmine bromide (PYR) have been used for the clinical treatment of myasthenia gravis and chronic heart failure [86, 87]. In addition, the development of new delivery methods (e.g., liposomes and nanoparticles) may reduce the side-effects of PYR [88, 89]. Evidence has shown that long-term treatment with PYR increases cardiac vagal tone, reduces sympathetic tone, and attenuates cardiac remodeling and left ventricular dysfunction during the progress of HF in mice [90]. More recent studies have demonstrated important roles for PYR in preserving autonomic balance [91, 92]. Our studies have also suggested that PYR restores baroreflex sensitivity, improves HRV, inhibits renin-angiotensin-system (RAS) activation and suppresses angiotensin II-induced cardiac fibroblast activation, thus ameliorating cardiac remodeling and improving peripheral vascular endothelial function in rats with myocardial infarctions or abdominal aortic constriction [17, 27, 93, 94]. These data support the notion that the enhancement of vagal tone by PYR is beneficial during cardiovascular disease. Seven days of PYR treatment has been demonstrated to improve intrinsic heart rate and autonomic tone, while reducing the area of myocardial infarction [95]. Yu *et al.* [96] have demonstrated that PYR treatment increases capillary density after myocardial infarction in rats. In addition, Durand *et al.* [97] have concluded that the immediate administration of this drug after myocardial infarction is an important pharmacological intervention to protect against potentially negative changes in cardiac autonomic imbalance. A recent report also indicated that PYR associated with exercise training benefits cardiovascular autonomic modulation and reduces inflammatory responses in infarcted rats [98]. Chronic treatment with PYR or donepezil increases cardiac vagal tone, reduces cardiomyocyte diameter, collagen density, and inflammatory cytokine levels in the plasma of SHRs [99]. Thus, the administration of acetylcholinesterase inhibitors may pharmacologically mimic activation of the cholinergic system, and contribute to cytokine production and the amelioration of myocardial infarction. The most recent study also demonstrated that the administration of PYR for 12 weeks ameliorates the cardiomyopathy induced by a high-fat diet in Sprague-Dawley rats, which is accompanied by improved vagal activity, reduced cardiac lipid accumulation, and the facilitated browning of white adipose tissue while activating brown adipose tissue [100]. However, studies are still necessary to better understand the pleiotropic effects of PYR on cardiovascular protection.

## Other Drugs for Cardiovascular Protection (Adenosine, Statins, β-Receptor Blockers, and ACEIs)

### Adenosine

Studies have demonstrated a functional link between adenosine and the vagal nerve activity [69]. Our study also demonstrated a possible functional interaction between muscarinic M2 receptors and α1 adenosine receptors in the I/R myocardium, and nitric oxide synthase may be the link between these two types of receptors [101]. Interestingly, adenosine has beneficial effects on M2 receptors, thus playing a role in the improvement of cardiac function. These results have demonstrated a possible new mechanism for the cardioprotection provided by adenosine. The precursor of adenosine (adenine sulfate) has also been suggested to have cardioprotective effects by increasing the expression of M2 receptors and cholinergic nerve density [102]. A recent study suggested that the inhibitory effects of VNS on HR and BP are partly mediated by endogenous adenosine release [103].

### Statins

Statin, the 3-hydroxy-3-methylglutaryl-CoA reductase inhibitor, is used routinely in coronary artery disease for its lipid-lowering effect. Evidence has indicated that the pleiotropic effects of statins may be involved in improving endothelial function, inhibiting inflammation and oxidative stress, attenuating myocardial remodeling, and stabilizing atherosclerotic plaques [104]. Our previous study demonstrated that atorvastatin enhances serum ACh levels and baroreflex sensitivity in I/R injury in rats [70]. In a human study, atorvastatin also had a beneficial impact on vagal activity, as measured by improvements in HRV, and might reduce the risk for arrhythmias in HF patients [105]. A recent study suggested that statins might reduce arrhythmias and improve HRV in healthy persons after 48 h of sleep deprivation [106]. The ejection fraction of HF patients without ischemic heart disease is preserved by stains [107]. However, the details of the underlying mechanisms of the effects of statins on cholinergic systems are still not fully clarified.

### β-Adrenoceptor Blockers

β-Blockers improve the prognoses of patients with HF. However, their protective effect is not fully dependent on the direct blockade of sympathetic activity. The sympathetic–parasympathetic interaction plays an important role in many cardiovascular diseases. The afferent sympathetic excitation results in activation of sympathetic efferent activity, together with the inhibition of vagal activity. These phenomena are antagonized by β-blockers [22]. β-ARs include three subtypes (β1, β2, and β3) [108]. Among them, β1 is primarily expressed in the heart. The cardiovascular protective effects exerted by β-blockers are due primarily to inhibition of the β1 receptor [109]. A previous study showed that carvedilol (an α- and β-blocker) increases the expression of M2 receptors in myocardium injured by adriamycin, implying that the up-regulation of these muscarinic receptors may be partly responsible for the protective effects of carvedilol in HF [71]. It has also been reported that long-term treatment with carvedilol restores autonomic tone and responsiveness in patients with moderate HF [110]. Vagal activation induced by metoprolol, another β-blocker, prevents ventricular fibrillation in mice with dilated cardiomyopathy [111].

### Angiotensin-Converting-Enzyme Inhibitors

Ramipril, an ACEI, increases HRV and cardiac function in patients with renal failure [112]. A recent study has also demonstrated that enalapril, combined with aerobic physical training, increases vagal tone [72]. In addition, delapril improves the sympatho-vagal balance and reduces atrioventricular blocks and ventricular arrhythmia [113]. Therefore, ACEIs might be a promising type of vagomimetic drug for the treatment of cardiovascular disease; however, clarifying the potential mechanism requires more studies.

## Conclusion

In this review, we have provided an overview of recent studies on the drug targets and vagomimetic drugs for vagus nerve activation in the treatment of cardiovascular disease (Table 1). This review shows that there has been a surge of data to suggest that decreased vagal activity is closely associated with the development of cardiovascular diseases and poor clinical outcomes. Targeting the vagus may be a promising therapeutic approach for the treatment of cardiovascular disease. Some vagomimetic drugs have been developed for the activation of vagal activity in cardiovascular diseases. The cardiovascular protective effects of these vagomimetic drugs may be predominantly correlated with a decrease in inflammation, the prevention of Ca^2+^ overload and the inhibition of RAS by improving vagal activity. The drugs discussed in this review all target vagal activation in cardiovascular diseases. Further study of these drug targets may help us to understand the mechanisms underlying vagal activation in these diseases.

**Table 1:** Drugs used in vagal nerve activation and potential mechanisms.

Classification	Drug	Mechanism	References
Cholinergic drugs	Choline	Increase of ACh levels	Liu, *et al.* 2017 [16]
		α7nAChR agonist	Lu, *et al.* 2015 [7]
		M3 agonist	Zhao, *et al.* 2010 [77]
		Inhibition of CaMKII and calcineurin	Wang, *et al.* 2012 [75]
		Inhibition of ROS	
	ACh	Down-regulation of AT1 receptor	Liu, *et al.* 2011 [79]
		Inhibition of ER stress	Bi, *et al.* 2015 [82]
		Inhibition of UPRmt	Xu, *et al.* 2016 [83]
		Inhibition of Ca^2+^ overload	He, *et al.* 2015 [84]
		M2/M3 agonist	Zhao, *et al.* 2017 [85]
AChE inhibitors	Pyridostigmine	AChE inhibition	Lataro, *et al.* 2015 [90]
	Donepezil	Anti-inflammation	Durand, *et al.* 2014 [91]
			Gavioli, *et al.* 2014 [92]
			Lu, *et al.* 2017 [27]
			Lu, *et al.* 2018 [100]
Nucleoside	Adenosine	Enhancement of cholinergic nerve density. Increase of M2 receptor expression	da Silva, *et al.* 2012 [69]
			Sun, *et al.* 2011[101]
			Jammes, *et al.* 2015 [103]
Statins	Simvastatin	Improvement of HRV	Millar, *et al.* 2014[104]
	Atorvastatin	Decrease of QT variability	Bi, *et al.* 2013 [70]
β-blockers	Carvedilol	Increase of M2 receptor expression	Xu, *et al.* 2006 [71]
	Metoprolol	improvement of HRV	Zhan, *et al.* 2009 [111]
ACEIs	Ramipril	Increase of HRV, cardiac function	Maida, *et al.* 2016 [72]
	Enalapril	Inhibition of RAS activation	Thireau, *et al.* 2015 [113]
	Delapril	Inhibition of Ca^2+^ overload	

ACh, acetylcholine; AChE, acetylcholine esterase; CaMKII, Ca^2+^ /calmodulin-dependent protein kinase II; ER, endoplasmic reticulum; HRV, heart rate variability; RAS, renin-angiotensin-system; ROS, reactive oxygen species; UPRmt, mitochondrial unfolded protein response.
